# Optimization of eco-friendly concrete with recycled coarse aggregates and rubber particles as sustainable industrial byproducts for construction practices

**DOI:** 10.1016/j.heliyon.2024.e25923

**Published:** 2024-02-12

**Authors:** Dhiraj Agrawal, Uday Waghe, Khalid Ansari, Mugahed Amran, Yaser Gamil, Ayed E. Alluqmani, Nitin Thakare

**Affiliations:** a Department of Civil Engineering, Yeshwantrao Chavan College of Engineering, Hingna Road, Wanadongri, Nagpur, 441110, India; b Department of Civil Engineering, College of Engineering, Prince Sattam Bin Abdulaziz University, 11942, Alkharj, Saudi Arabia; c Department of Civil Engineering, Faculty of Engineering and IT, Amran University, 9677, Amran, Yemen; d Department of Civil, Environmental and Natural Resources Engineering, Luleå University of Technology, Sweden; e Department of Civil Eng., School of Eng., Monash University Malaysia, Jalan Lagoon Selatan, 47500, Sunway, Selangor, Malaysia; f Department of Civil Engineering, Islamic University of Madinah, Madinah, 41411, Saudi Arabia; g Department of Civil Engineering, G. H. Raisoni Institute of Engineering and Technology, Nagpur, 441110, India

**Keywords:** Construction demolition waste, Waste tire rubber, RCA, Rubberized concrete

## Abstract

In this technology era, sustainable construction practices have become quite imperative. The exploration of alternative materials to reduce the environmental footprint is of paramount importance. This research paper delves into an exhaustive investigation concerning the utilization of recycled coarse aggregates (RCA) and rubber particles (RP) in concrete. It contributes to the growing body of knowledge aimed at fostering sustainable development in the construction industry by reducing waste, promoting recycling, and mitigating the environmental footprint of building materials. The objective of the study is to evaluate the potential benefits and limitations associated with incorporating these materials, thereby providing a sustainable alternative to conventional concrete. In this research, construction and demolition waste were recycled and used as RCA as a fractional switch of natural coarse aggregate (NCA) from 0% to 100%, with an increment of 20% replacement of NCA in concrete. The RP received from discarded tires generated as automobile industry waste were used as a volumetric fractional substitution of sand in concrete from 0% to 20%, with a 5% increment. No pre-treatment for RCA and RP was carried out before their utilization in concrete. A total of 26 mixes, including control concrete without NCA and RP, with a design strength of 40 MPa, were prepared and tested. Concrete mixes were examined for workability, density, mechanical, and durability properties. It was found that the concrete with 60% RCA and 10% RP showed satisfactory results in evaluation with the strength parameters of control concrete, as the compressive strength obtained for this concrete mix is 40.18 MPa, similar to the control mix. The optimization for RCA and RP was conducted using Response Surface Methodology (RSM). The major concern observed was a rise in water absorption with an increase in the percentage replacement of NCA and natural sand by RCA and RP. Findings from the investigation illustrate a promising prospect for the use of RCA and RP in concrete applications, displaying competent mechanical properties and enhanced durability under certain conditions, offering a viable option for environmentally friendly construction practices. However, the research also sheds light on some constraints and challenges, such as the variability in the quality of RCA and the necessity for meticulous quality control to ensure the reliability and consistency of the end product. It is discerned that further refinement in processing techniques and quality assurance measures is pivotal for mainstream adoption of RCA and RP in concrete construction.

## 1 Introduction

It is repoted that construction and demolition (C & D) waste contributes around 10–20% of municipal solid waste (MSW) production in India [1]. About 0.15 billion tons of C & D waste is produced in India annually, with a meager ratio of recycling as 6500 tons [2,3]. To deal with MSW generated by the construction industry, it is obligatory to think about recycling such C & D waste in the infrastructure industry itself to overcome the problem of landfilling and to conserve energy and natural resources used in the manufacturing of NCA [[4], [5], [6]]. At the same time, a remarkable upsurge is noted in the automobile industry in the last 4 decades, which in turn led to the generation of stockpiling of discarded tires. Annually around 1.6 billion fresh tires are produced and in contradiction, about 1 billion waste tires are disposed of around the globe [7]. A heavy mass of these waste tires is stockpiled or utilized in pyrolysis, these both processes are threatening to the environment [8,9]. To overcome the challenges raised due to waste tire generation its utilization in different segments are needed. Many researchers have tried and come up with promising results to use discarded tire particles in concrete and other infrastructural works as a substitution for coarse and fine aggregates to make lightweight concrete. In the present research work, the use of RCA and RP individually in concrete by many researchers was reviewed and the collective effect of RCA and RP on concrete was discussed after experimental work.

Chaudhary et al. [10] experimented on the utilization of C & D waste as RCA in concrete as a fractional substitution of natural aggregates. It is found that the 60% switch of coarse aggregates by RCA showed compressive strength more than the control concrete. The flexural properties were improved by almost 17.76% for a 60% substitution of NCA by RCA than the control concrete. In another research by Rahal [11], the water absorption of RCA was found to be higher by 3.47% than NCA. He also commented that the elastic modulus for concrete with RCA was reduced only by 3% when compared to NC, along with this the strains for compressive stress were higher than 5% for RCA concrete than the NCA concrete. McNeil, and Kang [12] also presented the outcome of RCA on the hardened properties of concrete. It is concluded that the compressive and flexural performance for concrete with RCA was less than NC, in addition to this it is noted that the elastic modulus was also decreased owing to the inclusion of RCA in concrete. It is stated that the weak interfacial bond between cement and demolished aggregates was a major cause of declined strengths. In the other experimental research by Martínez-Lage et al. [13], due to the water absorption and lightweight nature of RCA [14,15], the workability and density of RCA concrete are found to decline as the proportion of NCA replaced by RCA increases. The compressive strength was reduced by 30% for a 100% switch of NCA by RCA. The linear decrement is observed for the modulus of deformation of concrete with RCA in this study and it was around 40% for full replacement of NCA by RCA in concrete.

Lotfya and Al-Fayeza [16] researched concrete with RCA, it is replaced NCA up to 30% along with the replacement of sand with finer recycled aggregates. It is found that all the strength parameters have shown satisfactory results in comparison to control concrete [17]. It is recommended that the incorporation of RCA in concrete has also shown better performance for freezing-thawing resistance than the control concrete [18,19]. Also, Bravo et al. [20] completed research work on the use of RCA collected from different locations in concrete and commented that the common observation was seen in all forms of concrete with RCA as the strengths are decreased. The abrasion resistance of concrete is found to be enhanced with the inclusion of RCA in concrete. Zhou and Chen [21] experimented incorporation of RCA in concrete as fractional substitution of NCA, it is observed similar results of the decline in mass density and increment in water absorption due to the inclusion of RCA in concrete. It is also recorded the enhancement in compressive and flexural strength for concrete with RCA and there was a reduction in elastic modulus by incorporation of RCA in concrete. Another research work was performed by Ghorbel and Wardeh [22] on RCA concrete to determine the fracture properties and found that the RCA concrete mixes are more brittle than the control concrete. More loss was observed in fracture energy for RCA concrete due to the increased porosity of concrete caused by using RCA instead of NCA.

Bisht and Ramana [23], evaluated the durability of the RC, crumb rubber was used as a fractional exchange of fine aggregates up to 5.5%. It is seen that the mechanical strengths of concrete are decreased due to the incorporation of rubber. The maximum decrease is noted as 17.78% for 5.5% substitution to the control concrete, the flexural strength was decreased by 16.52%, and in contrast, the water penetration is boosted with a rise in rubber content. Ganjian et al. [24] studied the use of scrap tires in concrete by replacing coarse aggregates and cement with tire chips and tire powder with replacement levels of up to 10%. The substitution of 5% coarse aggregate by tire chips has resulted in enrichment of compressive strength and the replacement of cement showed a decrease in strength. Agrawal et al. [18] studied the effect of varying particle sizes of RP and the effect of pre-treatment opted on the mechanical properties of RC and concluded that the coarser size of RP has more promising results than the finer RP. Further, it is stated that the RC with 10% pretreated rubber fibers has almost similar results as that of the control concrete. Li et al. [25], also researched self-compacting concrete using rubber particles as aggregates, it is seen that the workability is reduced because of the fineness of RP and due to an increase in levels of replacement. At 30% substitution of sand for crumb rubber showed a decrement of 33.48% in comparison to control concrete. Authors [26] commented that the use of steel fibers in RC enhanced the flexural and tensile properties of RC as the steel fibers provide better bonding between cement paste and RP. The splitting tensile strength is observed to be enhanced by 21.56% for RC with 0.2% steel fibers and 10% RP than the control concrete, while the flexural strength is increased by almost 52% for the same mix in comparison to the control concrete.

Gholampour et al. [27] presented the results of an experiment on RC using crumb rubber from 0% to 18% by volume instead of sand. It is come up with the conclusion that the reduction in compressive strength and elastic modulus is reflected by increasing the amount of fine crumb rubber, the decline of 34.90% and 40.50% was recorded for compressive strength and static elastic modulus. Agrawal et al. [28] reviewed the effect of RP on the properties of geopolymer concrete (GPC) and concluded that the 30% fractional substitution for sand can be possible in GPC as beyond 30% switch drastic decline in mechanical properties is observed. Another study by Habib et al. [29] that replaced the coarse and fine aggregate with pretreated crumb rubber showed a reduction in workability by 17.28% and 20.98% for substitution levels of 15% and 25% respectively. The compressive strength was also decreased by 43.09% by the inclusion of 25% crumb rubber as a exchange of coarse and fine aggregate, in contradiction to this, the energy absorption capacity was increased significantly by 77% for a 15% replacement and damping ratio of concrete was improved by 91% for 25% substitution of aggregates by crumb rubber. The study [30] conducted on self-compacting concrete by Rajhans et al. concluded that the workability, compressive strength, and durability are reduced drastically with an increment in the amount of RCA in concrete. Further, it is also commented that the inclusion of silica fume along with RCA in concrete improved the mechanical properties of concrete [[31], [32], [33], [34]]. Also, Kisku et al. [35] conducted research on the microstructural investigation of concrete with RCA and commented that the use of RCA in concrete is a viable solution to environmental and disposal problems that arise from C & D Waste and also leads to making concrete sustainable.

Miller and Tehrani [36] replaced coarse aggregate with crumb rubber from 0% to 100% with an increment of 20% to make lightweight concrete, it is observed a decrease in compressive strength by 82.60% at 100% replacement of natural aggregates however, the flexural toughness was improved by 126.16% at 100% substitution. Su et al. [37] identified the properties of RC using uniform and varying sizes of crumb rubber as a fractional switch of sand and it is found a decrease in workability for finer rubber particles the maximum decrease in strengths was obtained for 0.3 mm RP. The amalgamation of all sizes of RP and their utilization in concrete showed a reduction in loss of strengths due to the appropriate gradation of RP. Also, Dong et al. [38] experimented on the RC using uncoated and coated RP as a partial switch for aggregates and this resulted in decrease in workability and compressive strength, the decrement was higher for uncoated RP as compared to the use of coated RP. The energy absorption was increased for RC in comparison to control concrete. Kardos and Durham [39], conducted study on RC with the usage of crumb rubber and RCA as substitution of fine and coarse aggregates respectively. To enhance the interfacial bonding between RP and cement particles, RP was pre-treated with silica fume. The study showed an improvement in workability and splitting tensile strength for 50% RCA and 20% RP. The elastic modulus was decreased by 33.33% for the same concrete mix.

Based on the literature reviews discussed, which have delved into the individual incorporation of RCAs and RPs in concrete, there remains a significant void in comprehensive research focusing on the synergistic effects and benefits of merging these materials for eco-friendly construction. This gap underscores the necessity for a detailed analysis, investigating the amalgamation of RCAs and RPs in concrete. This would further shed light on their collective impact on structural attributes and the environmental advantages, potentially pioneering sustainable building methods. Nevertheless, the study did meticulously assess the workability, compressive, split tensile, and flexural strength of the material. Moreover, tests for water absorption and acid resistance were conducted to evaluate the durability of the enhanced concrete.

## 2 Materials and methods

### 2.1 Materials

**Fig. 1:**
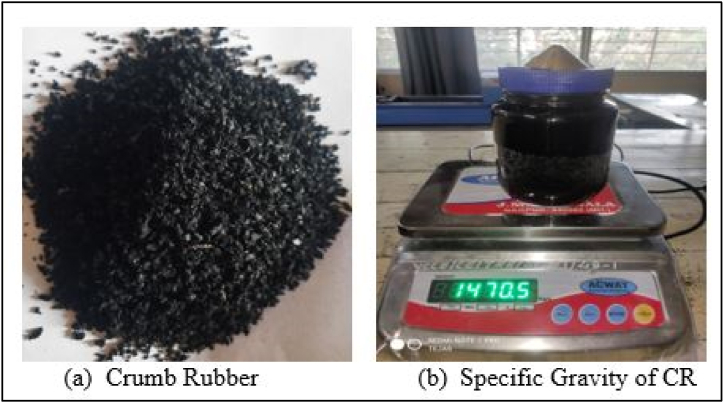
Crumb rubber sample and specific gravity of rubber particles.

**Fig. 2:**
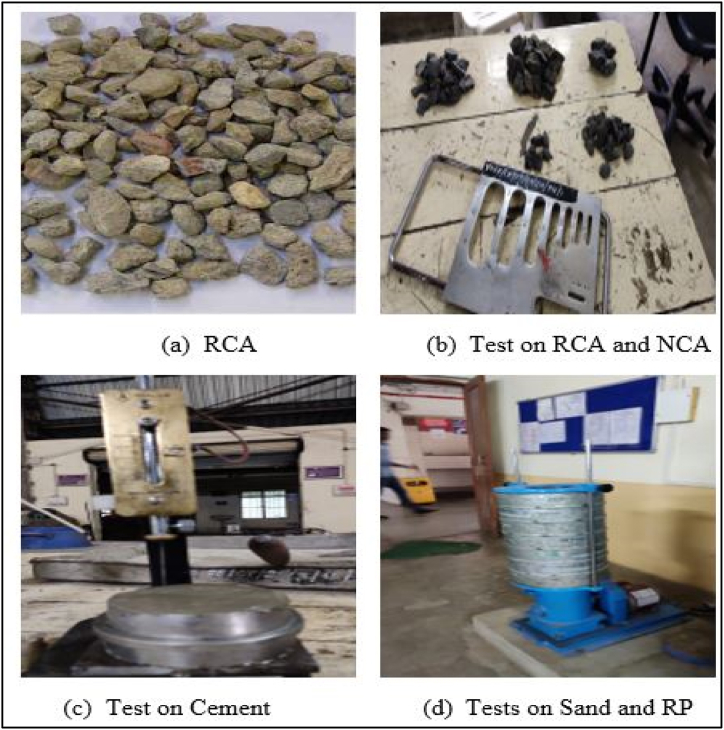
Tests performed on Aggregates of Concrete.

Ordinary Portland Cement (OPC) 43 grade with a specific gravity of 3.15 is used in this experiment. Zone II graded sand and well-graded angular coarse aggregates having a specific gravity of 2.65 and 2.74 correspondingly are used. RCA, as received from C & D waste with a specific gravity of 2.62, is used as a replacement for NCA. Crumb rubber of size up to 1.18 mm (Fig. 1(a–b) and a specific gravity of 1.02 is used as a partial substitution of sand from 0% to 20%, no pretreatment method is used for the crumb rubber. RCA received from wastes created in the demolition of concrete structures are directly used as a substitution of NCA from 0% to 100% for RCA the elongation and flakiness are checked and then implemented for replacement of NCA. For RCA no pretreatment was given to check its direct effect on the strength. FS and SF are used as cementitious materials, the specific gravity for both is 2.2. It is were used to maintain the permissible limits of OPC suggested by IS 456 [40]. All required preliminary tests were performed on the ingredients of concrete before computing the mix design calculations according to IS 10262-2019 [41]. Fig. 2(a and b) demonstrates the various tests performed on the aggregates of concrete.

### 2.2 Methods

This research paper employs a multifaceted approach to scrutinize the mechanical and environmental performance of concrete containing recycled coarse aggregates and rubber particles, focusing on assessing its suitability and efficacy for green construction. Extensive laboratory tests are conducted to evaluate the material properties, structural performance, and environmental impact, while real-world case studies are analyzed to glean insights into the practical applications and potential benefits of utilizing such sustainable materials in the construction industry. The 26 various mixes of concrete were prepared in this experiment, the quantity of materials used for preparing concrete mixes is explained in Table 1.

**Table 1:** Mix Proportion of concrete mixes in kg for 1 m^3^ Concrete.

SN	Mix	Cement	Fly Ash	Silica Fume	Water	NCA	FA	RP	RCA	W/C Ratio	Density
1	0RC0RP	445	84	34	225	1006	538	0	0	0.4	2332
2	20RC0RP	445	84	34	225	805	538	0	192	0.4	2323
3	40RC0RP	445	84	34	225	604	538	0	385	0.4	2315
4	60RC0RP	445	84	34	225	402	538	0	577	0.4	2305
5	80RC0RP	445	84	34	225	201	538	0	770	0.4	2297
6	100RC0RP	445	84	34	225	0	538	0	962	0.4	2288
7	20RC5RP	445	84	34	225	805	511	10	192	0.4	2306
8	40RC5RP	445	84	34	225	604	511	10	385	0.4	2298
9	60RC5RP	445	84	34	225	402	511	10	577	0.4	2288
10	80RC5RP	445	84	34	225	201	511	10	770	0.4	2280
11	100RC5RP	445	84	34	225	0	511	10	962	0.4	2271
12	20RC10RP	445	84	34	225	805	484	21	192	0.4	2290
13	40RC10RP	445	84	34	225	604	484	21	385	0.4	2282
14	60RC10RP	445	84	34	225	402	484	21	577	0.4	2272
15	80RC10RP	445	84	34	225	201	484	21	770	0.4	2264
16	100RC10RP	445	84	34	225	0	484	21	962	0.4	2255
17	20RC15RP	445	84	34	225	805	457	31	192	0.4	2273
18	40RC15RP	445	84	34	225	604	457	31	385	0.4	2265
19	60RC15RP	445	84	34	225	402	457	31	577	0.4	2255
20	80RC15RP	445	84	34	225	201	457	31	770	0.4	2247
21	100RC15RP	445	84	34	225	0	457	31	962	0.4	2238
22	20RC20RP	445	84	34	225	805	430	41	192	0.4	2256
23	40RC20RP	445	84	34	225	604	430	41	385	0.4	2248
24	60RC20RP	445	84	34	225	402	430	41	577	0.4	2238
25	80RC20RP	445	84	34	225	201	430	41	770	0.4	2230
26	100RC20RP	445	84	34	225	0	430	41	962	0.4	2221

### 2.3 Experimental tests setup and procedures

The fresh properties of concrete like fresh density and workability are determined. Fresh and hardened properties of RC are computed to check the effect of RCA and RP in comparison to control concrete with 0% RCA and 0% RP.

#### 2.3.1 Fresh density

The fresh density of control concrete and RC were calculated for all mixes by referring to ASTM C138 [42]. The workability was evaluated using slump cone apparatus IS 1199 [43].

#### 2.3.2 Hardened properties of concrete

**Fig. 3:**
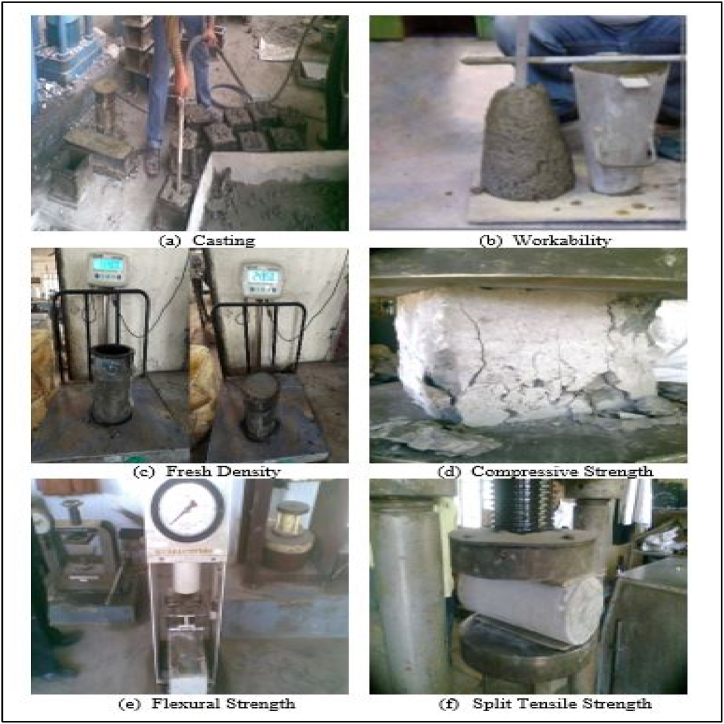
Casting and testing of concrete.

Concrete cubes of 150 mm were tested on compressive testing equipment with a capacity of 2000 kN after 28 days of cure. Under a two-point loading flexural testing equipment, the flexural test was carried out on rectangular beams with a length of 500 mm and a cross-section of 100 mm × 100 mm. The splitting tensile strength was determined using concrete cylinders that were 300 mm long and 150 mm in diameter. All these tests were conducted by referring to IS 516 [44]. Fig. 3(a–f) shows the testing processes for all specimens to evaluate the properties of concrete.

#### 2.3.3 Durability properties of concrete

**Fig. 4:**
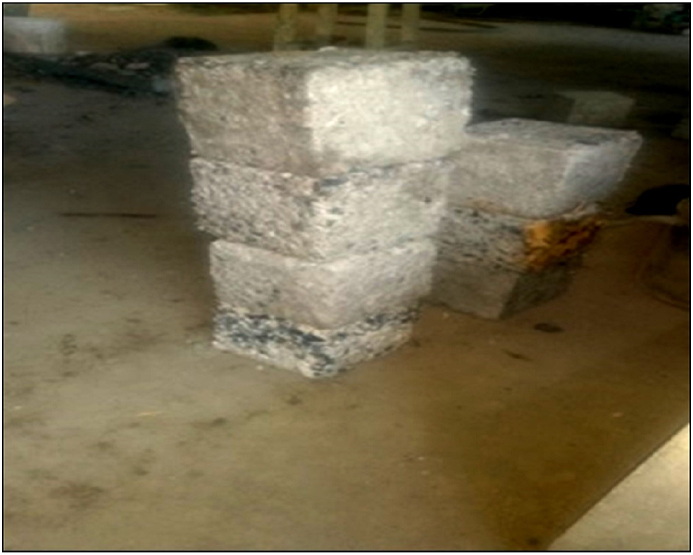
Concrete specimens after acidic action.

Along with the mechanical properties, the concrete needs to be tested for its durability. To check the durability of modified concrete with the incorporation of RCA and RP, RC mixes and control concrete specimens were evaluated for their water absorption and acid resistance. The water absorption of concrete mixes was done according to the BS 1881 [45] and the acid attack test was conducted by referring to ASTM C1898 [46]. The specimens used for the water absorption test were of size 75 × 75 × mm correction factor for volume change was maintained as 1. The acid attack test was conducted on cubes of size 150 × 150 × 150 mm. The concrete cubes were first cured for 28 days in water and then immersed in sulfuric acid by maintaining a pH of 2. The compressive strength of cubes was compared after a chemical attack with the specimens cured for 28 days. The effect of acid attack on concrete specimens is shown in Fig. 4. The.

## 3 Results and discussions

### 3.1 Density of fresh concrete

The freshly prepared concrete mixes of different proportions were used to evaluate the fresh density of concrete according to the standards explained in ASTM C138 [42], the cylinder of known volume was used and its empty weight was taken after that cylinder was filled with concrete and its weight is determined. The difference between this weight and the empty weight of the cylinder is divided by the volume of the cylinder to calculate the fresh density.

**Fig. 5:**
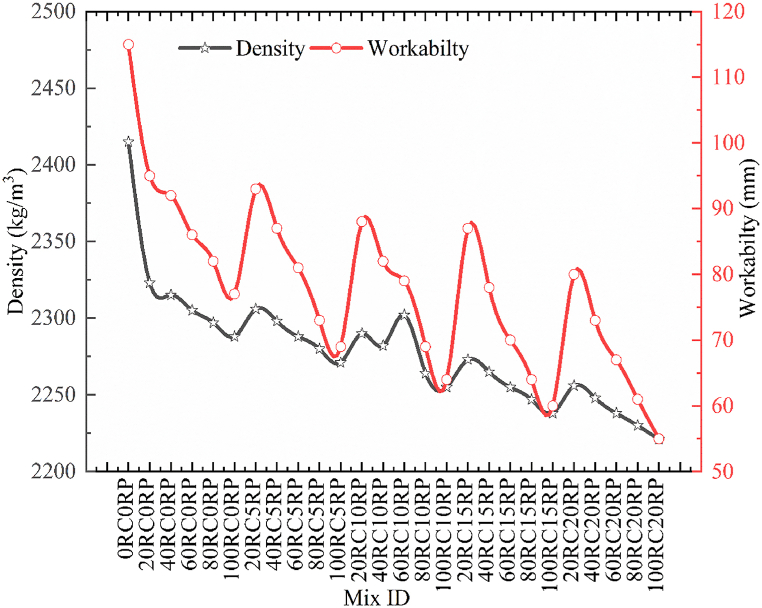
Density and workability for concrete mixes.

The decline in the density of RC was observed by an upsurge in the amount of substitution of NCA by RCA and sand by RP. A maximum decrease of 8.03% was noted for the 100RC20RP concrete mix. The maximum density was recorded for control concrete as 2415 kg/m^3^. Similar results for the decrement in density were obtained by other researches [23,25] in their experimental studies. Fig. 5 represents the variation in fresh densities for all concrete mixes.

### 3.2 Workability

To determine another fresh concrete property, the workability is checked by following IS 1199 [43]. The vessel of a height of 300 mm and top and bottom diameters of 100 and 200 mm respectively. Concrete was poured in three layers compacting every layer then after removing the vessel the slump was measured the workability of the concrete was found to be decreased, almost 52.17% decrease was noted for the workability of 100RC20RP when linked to control concrete. To check the actual effect of RCA and RP no pre-treatment is adopted for both waste materials before their incorporation into concrete. The cause for this remarkable decrease in a slump was the water absorption tendency of RCA and RP. The study found that workability can be maintained using chemical admixtures (Fig. 5). Similarly, Habib et al. and Rahal [11,29] suggested using high range water reducing agents to maintain the desired workability.

### 3.3 Compressive strength

**Fig. 6:**
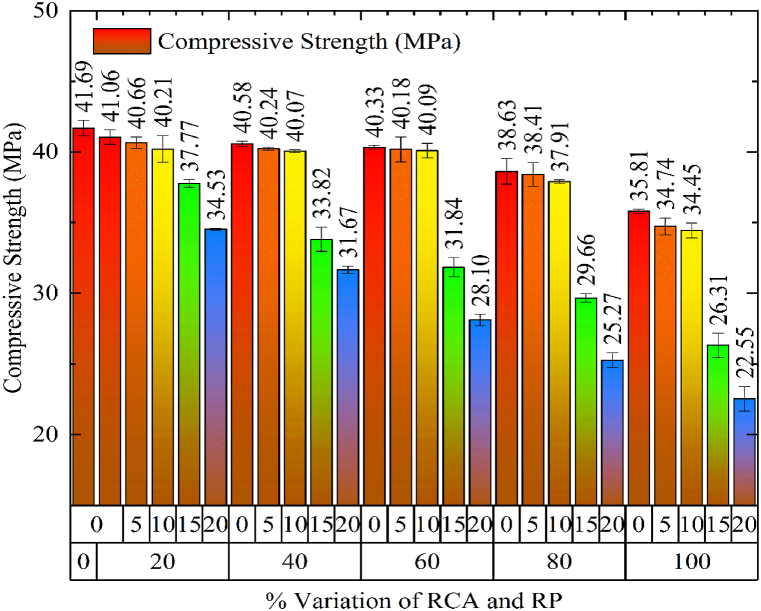
Comparison of Compressive Strength for varying percentages of RCA and RP.

According to IS 516 [44], the compressive strength of distinct mixes was assessed and compared with control concrete. After curing for 28 days, the compressive strength for each specimen was computed and an average of three samples was taken to show its compressive strength. A gradual drop was noted in compressive strength with the substitution of NCA and sand by RCA and RP (Fig. 6). The rate of reduction for compressive strength was increased after 60% inclusion of RCA and sand replacement beyond 10%. The maximum decrease of 45.91% was observed for concrete mix with 100% RCA and 20% RP. However, the concrete mix 60RC10RP showed 40.18 MPa strength, which is required as per the design mix. The maximum decrease of compressive strength is noted as 45.91% for 100RC20RP in comparison to the control concrete. The inclusion of RP in the concrete leads to weak interfacial bonding amongst cement and rubber particles which in turn lessens the compressive strength.

### 3.4 Flexural strength and split tensile strength

**Fig. 7:**
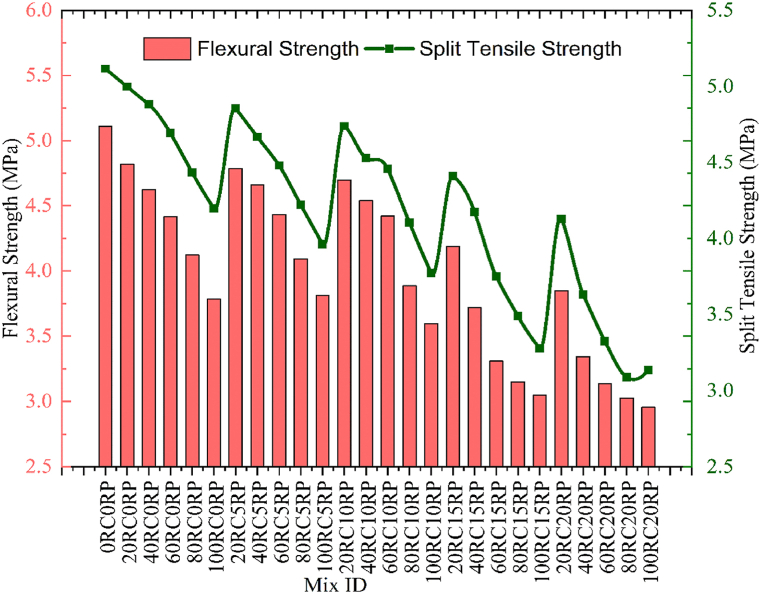
Comparison of flexural and split tensile strength for concrete.

The flexural strength of concrete was determined by referring to IS 516 [44], the rectangular beams of size 100 × 100 × 500 mm were tested after curing. The average of three samples was computed to fix the flexural strength of a particular concrete mix. A gradual decrease in flexural strength was noted for all mixes. Replacement of NCA by RCA has reflected in a decrement in the strength. Fig. 7 demonstrates the variation in flexural strength owing to the inclusion of RCA and RP in concrete. The minimum flexural strength was observed for mix 100RC20RP, the strength was decreased by 42.14% as compared to the control concrete.

The split tensile strength was also computed about IS 516 [44]. Cylindrical specimens were tested after 28 days of curing. By referring to Fig. 7 it is observed the decline ratio of split tensile strength is quite less than that of reduction in flexural strength. A maximum decrease of 38.71% was observed for the 100RC20RP concrete mix. The inclusion of RCA has affected split tensile strength adversely resulting in a decrease in strength.

### 3.5 Water absorption test

**Fig. 8:**
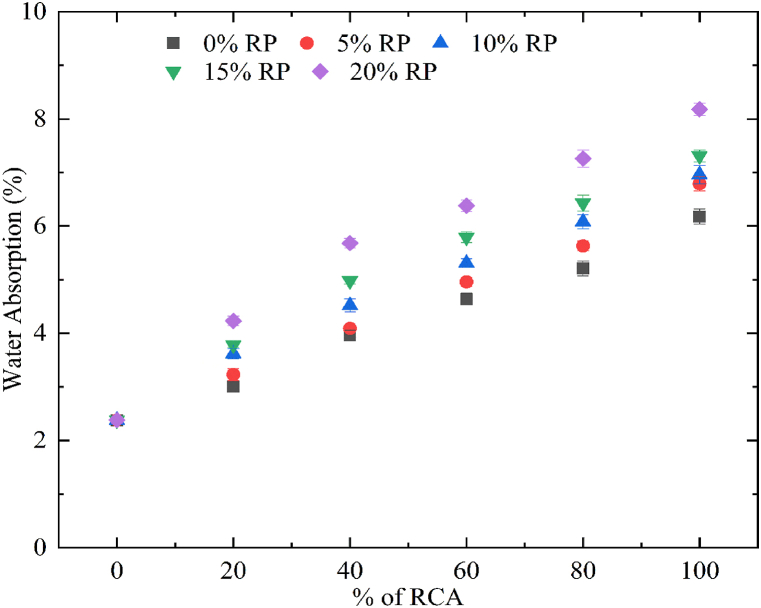
Variation in water absorption of concrete.

By observing the results of RC for mechanical properties, the weak bonding between cement paste, RCA, and RP was recorded as a decrease in compressive strength was noted. The water absorption of RC mixes was found to be increased linearly with the incorporation of RCA and RP. The water absorption of the control concrete was recorded as 2.38% while the maximum water absorption was noted for the mix 100RC20RP as 8.18%. The water absorption for concrete mix with 100% RCA as replacement of NCA was 1.6 times higher than the control concrete. Fig. 8 demonstrates the results obtained for water absorption for all concrete mixes. The inclusion of RP in RC also showed an upsurge in water absorption. The amalgamation of RP from 5% to 20% with an increment of 5% in concrete mixes increased the water absorption by 1.85, 1.92, 2.07, and 2.43 times the water absorption of control concrete subsequently for 100% substitution of NCA using RCA. The inclusion of RCA in RC mixes has been affected more adversely than the incorporation of RP. The tendency of RCA and RP to absorb water and the voids generated owing to the weak bonding between cement paste, RCA, and RP resulted in increased water absorption. A similar increase in water absorption for concrete mixes RCA was noted by previous experimental works [47].

### 3.6 Acid attack test

**Fig. 9:**
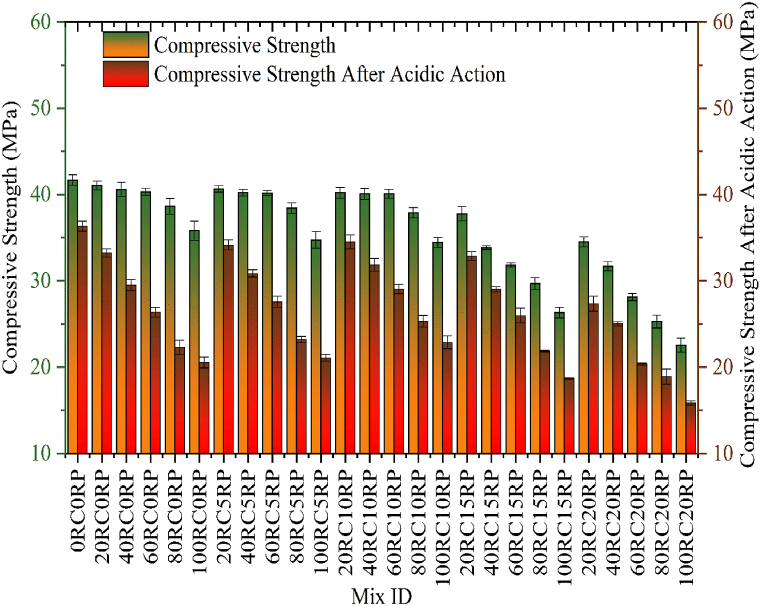
Variation in compressive strength of concrete mixes for acid attack test.

The acid attack test on all concrete specimens was assessed by following the ASTM standards [46]. The acid attack test was conducted on all concrete specimens and compared with the compressive strength of each specimen after curing for 28 days (Fig. 9). The concrete specimens were immersed in the sulfuric acid solution having a pH of 2 for 56 days. The results of the acid attack revealed that the compressive strength for all RC mixes was decreased with an increase in the amount of RCA and RP. The decrease in compressive strength of RC mixes was more in the concrete mixes with higher amounts of RCA. The bonding between cement paste and the aggregates of concrete is decreased due to acid attack and crack formation is noted. The modified concrete mixes with RP up to 10% showed less depth of propagation of cracks as compared to the control concrete. The effect of the inclusion of RP as a partial switch of sand lessened the reduction ratio slightly up to 10%. Further replacement of sand using RP decreased the strength drastically. The maximum decrease was observed for the mix 100RC0RP as the compressive strength was lessened by 42.64% when compared to the 28-day compressive strength of the same mix. Considering the 28-day compressive strength of the control mix the maximum decrease in compressive strength was observed as 61.98% for the mix 100RC20RP. For the RC mix 60RC10RP having satisfactory results for the mechanical properties the decline of 30.31% was noted for acidic action.

### 3.7 Optimization using response surface methodology

After determining the mechanical properties of all concrete mixes. Response Surface Methodology (RSM) opted to perform the statistical analysis for optimizing the amounts of replacement of NCA and sand using RCA and RP. Central Composite Design (CCD) was used for analysis [17,19,48]. The RCA and RP were considered as factors for optimization and the compressive strength, flexural strength, and split tensile strength were considered as the responses. The quartic design models were generated for compressive, flexural, and split tensile strengths respectively. Table 2 and Table 3 demonstrate the fit summary and Analysis of Variance (ANOVA) results for all responses with 26 runs used in this current study. Equations (1), (2), and (3) address the compressive, flexural, and splitting tensile strengths after regression analysis (Table 4). A strong correlation was noted with R squared (R2) values as 0.9934, 0.9926, and 0.9963 for compressive, flexural, and splitting tensile strength respectively [49].

**Table 2:** Actual and predicted interaction of various outcome levels in MPa.

Run	Factor 1 A: RCA%	Factor 2 B: RP%	Response 1		Response 2		Response 3	
			Compressive Strength		Flexural Strength		Splitting Tensile Strength	
			Actual	Predicted	Actual	Predicted	Actual	Predicted
1	0	0	41.69	41.74	5.11	5.11	5.12	5.12
2	20	0	41.06	40.98	4.82	4.79	5.00	5.01
3	40	0	40.58	40.73	4.62	4.66	4.88	4.87
4	60	0	40.33	40.29	4.42	4.44	4.69	4.68
5	80	0	38.63	38.85	4.12	4.09	4.43	4.44
6	100	0	35.81	35.51	3.79	3.80	4.20	4.20
7	20	5	40.66	40.19	4.79	4.79	4.86	4.79
8	40	5	40.24	40.25	4.66	4.65	4.67	4.69
9	60	5	40.18	39.94	4.43	4.44	4.48	4.51
10	80	5	38.41	38.56	4.09	4.11	4.22	4.24
11	100	5	34.74	35.29	3.81	3.80	3.96	3.95
12	20	10	40.21	41.17	4.70	4.77	4.74	4.77
13	40	10	40.07	40.43	4.54	4.52	4.53	4.61
14	60	10	40.09	39.44	4.42	4.28	4.46	4.37
15	80	10	37.91	37.57	3.89	3.94	4.10	4.07
16	100	10	34.45	34.12	3.60	3.64	3.77	3.78
17	20	15	37.77	36.56	4.19	4.12	4.41	4.41
18	40	15	33.82	34.36	3.72	3.72	4.17	4.10
19	60	15	31.84	32.28	3.31	3.45	3.75	3.79
20	80	15	29.66	29.83	3.15	3.17	3.49	3.50
21	100	15	26.31	26.38	3.05	2.96	3.28	3.29
22	20	20	34.53	35.06	3.85	3.87	4.13	4.12
23	40	20	31.67	31.15	3.34	3.35	3.63	3.65
24	60	20	28.10	28.06	3.14	3.11	3.32	3.30
25	80	20	25.27	25.35	3.03	2.98	3.09	3.10
26	100	20	22.55	22.51	2.96	3.01	3.14	3.12

**Table 3:** Results of ANOVA for all responses.

Source	Sum of Squares	df	Mean Square	F-value	p-value	
**Compressive Strength**						
**Model**	756.54	14	54.04	118.46	<0.0001	significant
A-RCA	5.12	1	5.12	11.23	0.0065	
B-RP	56.04	1	56.04	122.84	<0.0001	
AB	6.51	1	6.51	14.27	0.0031	
A^2^	0.4884	1	0.4884	1.07	0.3230	
B^2^	30.15	1	30.15	66.09	<0.0001	
A^2^B	1.14	1	1.14	2.50	0.1423	
AB^2^	3.11	1	3.11	6.81	0.0243	
A³	0.4363	1	0.4363	0.9563	0.3491	
B³	3.10	1	3.10	6.80	0.0244	
A^2^B^2^	0.9962	1	0.9962	2.18	0.1675	
A³B	0.0419	1	0.0419	0.0919	0.7674	
AB³	756.54	14	54.04	118.46	<0.0001	
A⁴	5.12	1	5.12	11.23	0.0065	
B⁴	56.04	1	56.04	122.84	<0.0001	
**Residual**	6.51	1	6.51	14.27	0.0031	
**Cor Total**	0.4884	1	0.4884	1.07	0.3230	
**Flexural Strength**						
**Model**	10.32	14	0.7368	105.75	<0.0001	significant
A-RCA	0.3024	1	0.3024	43.40	<0.0001	
B-RP	1.22	1	1.22	175.02	<0.0001	
AB	0.0122	1	0.0122	1.76	0.2120	
A^2^	0.0121	1	0.0121	1.73	0.2148	
B^2^	0.3156	1	0.3156	45.29	<0.0001	
A^2^B	0.0705	1	0.0705	10.13	0.0087	
AB^2^	0.0005	1	0.0005	0.0650	0.8035	
A³	0.0129	1	0.0129	1.85	0.2015	
B³	0.1595	1	0.1595	22.89	0.0006	
A^2^B^2^	0.0205	1	0.0205	2.94	0.1143	
A³B	0.0045	1	0.0045	0.6524	0.4364	
AB³	0.0129	1	0.0129	1.85	0.2009	
A⁴	0.0133	1	0.0133	1.91	0.1939	
B⁴	0.1891	1	0.1891	27.15	0.0003	
**Residual**	0.0766	11	0.0070			
**Cor Total**	10.39	25				
**Splitting Tensile Strength**						
**Model**	8.70	14	0.6211	209.95	<0.0001	significant
A-RCA	0.3154	1	0.3154	106.61	<0.0001	
B-RP	0.4698	1	0.4698	158.80	<0.0001	
AB	0.0342	1	0.0342	11.57	0.0059	
A^2^	0.0090	1	0.0090	3.03	0.1096	
B^2^	0.1318	1	0.1318	44.55	<0.0001	
A^2^B	0.0277	1	0.0277	9.38	0.0108	
AB^2^	0.0053	1	0.0053	1.79	0.2081	
A³	0.0005	1	0.0005	0.1539	0.7023	
B³	0.0001	1	0.0001	0.0189	0.8930	
A^2^B^2^	0.0368	1	0.0368	12.43	0.0047	
A³B	0.0001	1	0.0001	0.0331	0.8588	
AB³	0.0086	1	0.0086	2.91	0.1159	
A⁴	0.0003	1	0.0003	0.1162	0.7397	
B⁴	0.0699	1	0.0699	23.63	0.0005	
**Residual**	0.0325	11	0.0030			
**Cor Total**	8.73	25

**Table 4:** Equations from regression analysis for all responses.

Response	Regression Model	Equation
Compressive Strength	= +40.00–2.43 × A −7.29 × B - 4.80 × AB - 1.70 × A^2^ - 15.66 × B^2^ + 1.86 × A^2^B - 1.93 × AB^2^ - 1.59 × A³ + 1.76 × B³ + 1.77 × A^2^B^2^ + 0.4985 × A³B +1.46 × AB³ - 0.1635 × A⁴ + 10.71 × B⁴	(1)
Flexural Strength	= +4.41–0.5902 × A - 1.08 × B - 0.2080 × AB - 0.2673 × A^2^ - 1.60 × B^2^ + 0.4638 × A^2^B + 0.0233 × AB^2^ - 0.2730 × A³ + 0.3991 × B³ + 0.2536 × A^2^B^2^ - 0.1642 × A³B + 0.1891 × AB³ + 0.3668 × A⁴ + 1.08 × B⁴	(2)
Splitting Tensile Strength	= +4.50–0.6028 × A - 0.6673 × B - 0.3479 × AB - 0.2303 × A^2^ - 1.04 × B^2^ + 0.2909 × A^2^B - 0.0798 × AB^2^ + 0.0514 × A³ + 0.0075 × B³ + 0.3397 × A^2^B^2^ + 0.0241 × A³B + 0.1546 × AB³ + 0.0589 × A⁴ + 0.6595 × B⁴	(3)

**Fig. 10:**
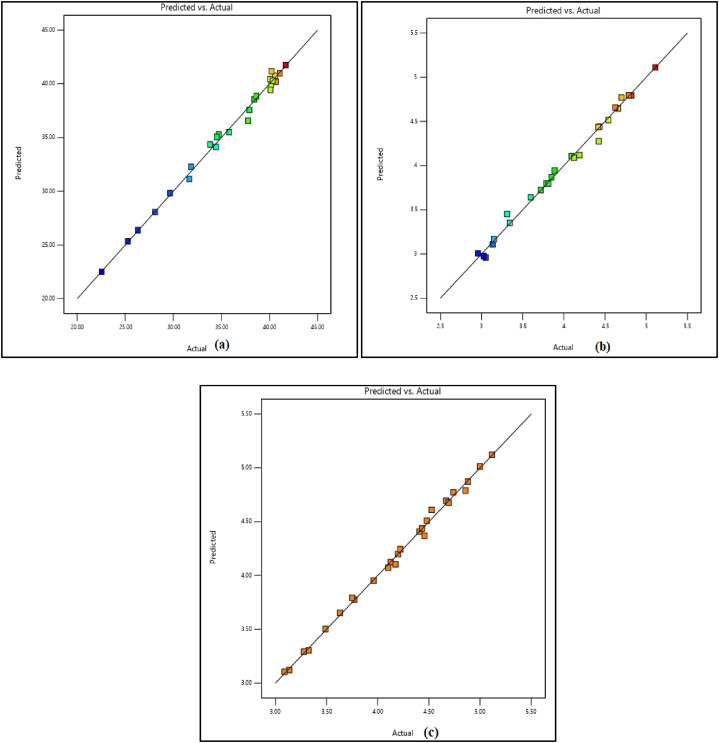
Predicted strength versus actual strength for (a) compressive strength, (b) flexural strength, and (c) splitting tensile strength.

**Fig. 11:**
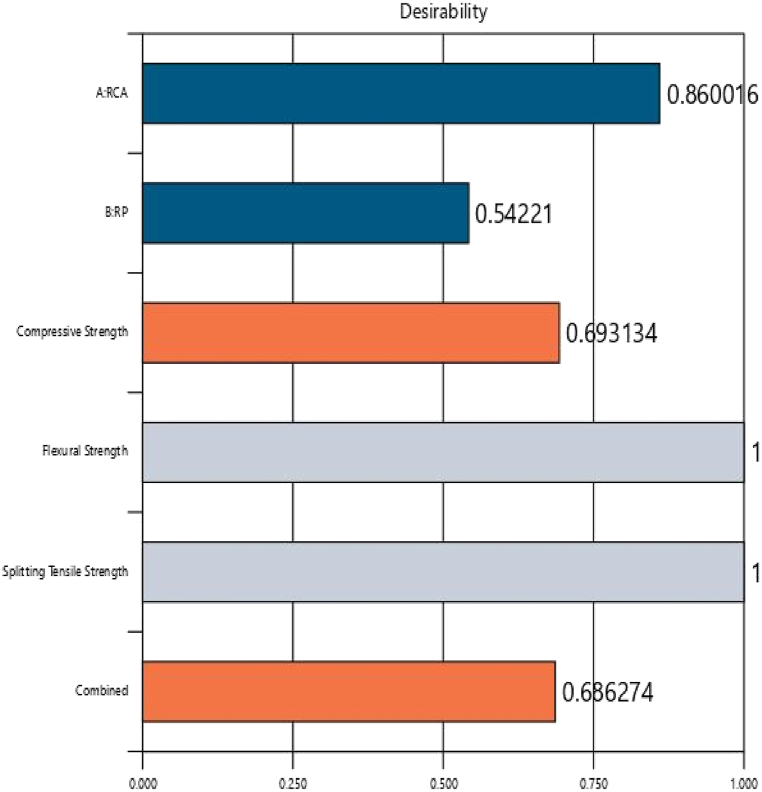
Desirability chart for optimized combination.

**Fig. 12:**
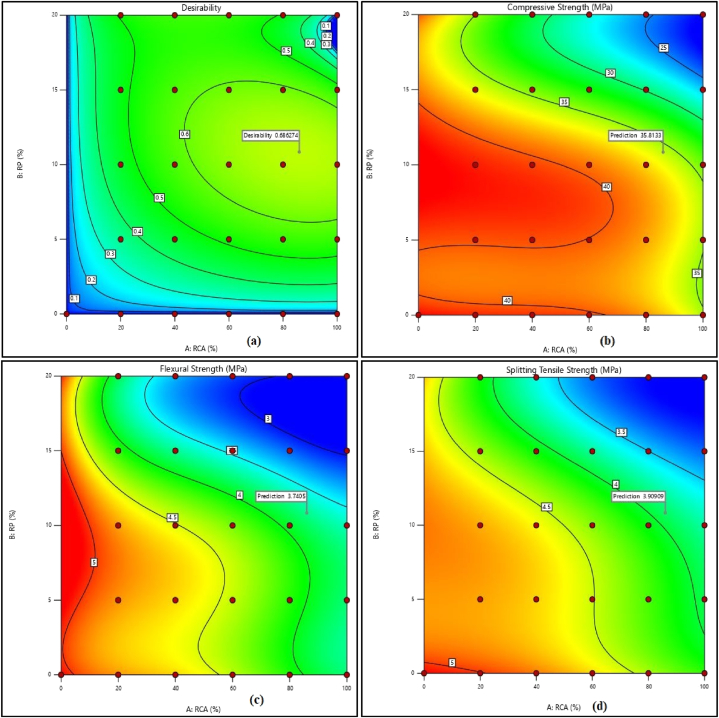
Contour map for (a) desirability, (b) compressive strength, (c) flexural strength, and (d) splitting tensile strength for optimized combination.

Fig. 10(a–c) depicts the predicted versus actual results for all responses. The solution obtained from the analysis and optimization using the RSM technique showed that the optimum replacement possible for NCA using RCA was up to 86% with 10.84% RP. The compressive, flexural, and splitting tensile strengths obtained for this combination were 35.81 MPa, 3.74 MPa, and 3.91 MPa respectively. The desirability achieved for this combination was 0.686 (Fig. 10.) The contour plots for the optimized combination are shown in Fig. 11 while Fig. 12(a–d) exhibits contour map for desirability and strength properties for optimized combination.

## 4 Conclusions

In this study, the focused efforts have been to navigate towards the culmination of this experimental investigation and response surface methodology (RSM) studies. It is pivotal to distill the essence of the findings and reflect on the implications it is hold for the realm of sustainable construction. The research has traversed the intricate landscape of incorporating recycled coarse aggregates (RCA) and rubber particles (RP) in concrete. This has unveiled a spectrum of possibilities and challenges that warrant thoughtful consideration and future exploration.

The study aimed to evaluate the potential benefits and limitations of incorporating RCA and RP into concrete, seeking to offer a sustainable alternative to conventional concrete. The research focused on promoting recycling, reducing waste, and mitigating the environmental footprint of building materials by leveraging construction and demolition waste. The excerpt provides more details on the methodology employed in the research. The study experimented with using RCA as a partial replacement for natural coarse aggregate (NCA) in concrete, with replacement levels ranging from 0% to 100% in increments of 20%. Also, RP, derived from discarded tires, were used as a volumetric fractional substitution for sand in concrete, with substitution levels ranging from 0% to 20% in increments of 5%. This paper delves into the results of the experiments conducted on the concrete mixes. A total of 26 mixes were prepared and tested for workability, density, mechanical, and durability properties. The results indicated that a concrete mix with 60% RCA and 10% RP showed satisfactory results compared to control concrete, achieving a compressive strength of 40.18 MPa, similar to the control mix. The optimization for RCA and RP was conducted using RSM.

− Moreover, a nuanced observation revealed an increase in water absorption with a heightened percentage of RCA and RP, indicating a trade-off between sustainability and material properties. Despite this, the promising mechanical properties and enhanced durability under specific conditions showcased by the optimized mix underscore the feasibility of integrating RCA and RP in construction, offering a tangible solution to mitigate the environmental footprint of building materials. However, the following conclusions were formed based on the findings and conversations.
− The water absorption by RCA and RP resulted in a reduction of the workability of concrete drastically. Water absorption of RCA was more and hence workability is reduced almost by 43.18%.
− As the density of RP is much less compared to the sand the density of RC is reduced and it suggests that the lightweight concrete can be prepared using RP.
− The weaker interfacial bonding between RCA, cement paste, and RP due to the smoothness of RP and RCA, has reduced the strength of RC. The concrete mix 60RC10RP has shown a satisfactory compressive strength to that of the control concrete.
− Flexural and split tensile strength is mainly reduced due to the substitution of NCA with RCA. As rubber is an elastic material the reduction rate for flexural and split tensile strength for replacement ratios of RP was minimal.
− The water absorption of RC mixes is increased as compared to the control concrete and affects the durability of modified concrete mixes. The water absorption of RC mixes up to the replacement levels of 60% for RCA and 10% for RP showed satisfactory results.
− The reduction in compressive strength owing to acid attack is found to be more for the replacement of NCA using RCA than the substitution of sand using RP.
− The RSM analysis showed that the 86% RCA can be utilized as a substitution for NCA and 10.84% RP can be used as a fractional switch of sand with a desirability coefficient of 0.686.
− It is recommended to give pre-treatment to RCA and RP before its utilization in concrete, to enhance the properties. RCA can be soaked in water before its application in concrete to maintain the workability of modified concrete.

While this investigation sheds light on the promising prospects of RCA and RP in concrete applications, it also uncovers the layers of complexity associated with material variability and challenges in balancing sustainability with performance. This research, therefore, serves as a foundation for further exploration and refinement in the application of recycled materials in construction. Future studies could delve deeper into addressing the identified constraints, optimizing material properties, and expanding the application spectrum of RCA and RP in the construction industry.

In conclusion, the insights garnered from this study pave the way for a paradigm shift in construction practices, fostering sustainability, and reducing waste. The amalgamation of RCA and RP in concrete manifests as a beacon of hope for environmentally conscious construction, albeit with considerations for the inherent challenges and opportunities for further advancement.

## CRediT authorship contribution statement

**Dhiraj Agrawal:** Writing – original draft, Methodology, Investigation, Formal analysis, Data curation, Conceptualization. **Uday Waghe:** Writing – original draft, Software, Methodology, Investigation, Formal analysis, Conceptualization. **Khalid Ansari:** Writing – original draft, Software, Methodology, Investigation, Formal analysis, Data curation, Conceptualization. **Mugahed Amran:** Writing – review & editing, Visualization, Validation, Resources, Funding acquisition, Formal analysis, Data curation. **Yaser Gamil:** Writing – review & editing, Visualization, Validation, Resources, Funding acquisition, Formal analysis. **Ayed E. Alluqmani:** Writing – review & editing, Visualization, Validation, Resources, Formal analysis, Data curation. **Nitin Thakare:** Writing – review & editing, Visualization, Validation, Data curation.

## Declaration of competing interest

The authors declare that they have no known competing financial interests or personal relationships that could have appeared to influence the work reported in this paper.

## Abbreviations

RCA: Recycled coarse aggregate
C & D: Construction and Demolition Waste
FA: Fine Aggregate
FS: Fly Ash
MSW: Municipal Solid Waste
NCA: Natural coarse aggregate
RC: Rubberized Concrete
RP: Rubber particles
SF: Silica Fume

## References

[1] (2016). CPHEEO municipal solid waste management manual Part II. Minist. Urban Dev..

[2] sukanya.nair@cseindia.org Construction and Demolition Waste.

[3] da Silva T.R., de Azevedo, Cecchin D., Marvila M.T., Amran M., Fediuk R., Vatin N., Karelina M., Klyuev S., Szelag M., A.R.G (2021). Application of plastic wastes in construction materials: a review using the concept of life-cycle assessment in the context of recent research for future perspectives. Materials.

[4] Amran M., Onaizi A.M., Qader D.N., Murali G. (2022).

[5] Lesovika V.S., Ahmed A.A., Fediuk R.S., Kozlenko B., Amran Y.H.M., Alaskhanov A.K., Asaad M.A., Murali G., Uvarov V.A. (2021). Performance investigation of demolition wastes-based concrete composites. Mag. Civ. Eng..

[6] Chin W.Q., Lee Y.H., Amran M., Fediuk R., Vatin N., Kueh A.B.H., Lee Y.Y. (2022). A sustainable reuse of agro-industrial wastes into green cement bricks. Materials.

[7] Goldstein Market Intelligence Global Tire Industry Analysis by Product Type and, by Geography & COVID-19 Impact with Market Outlook 2017–2030.

[8] Siddika A., Mamun M.A. Al, Alyousef R., Amran Y.H.M., Aslani F., Alabduljabbar H. (2019). Properties and utilizations of waste tire rubber in concrete: a review. Construct. Build. Mater..

[9] Al-Fakih A., Mohammed B.S., Wahab M.M.A., Liew M.S., Mugahed Amran Y.H., Alyousef R., Alabduljabbar H. (2020). Characteristic compressive strength correlation of rubberized concrete interlocking masonry wall. Structures.

[10] Chaudhary B., Dhiman S., Talwar R., Arif S.M., Verma V. (2021). Experimental investigation of strength of concrete using recycled demolished construction materials as coarse aggregate. Mater. Today Proc..

[11] Rahal K. (2007). Mechanical properties of concrete with recycled coarse aggregate. Build. Environ..

[12] McNeil K., Kang T.H.K. (2013). Recycled concrete aggregates: a review. Int. J. Concr. Struct. Mater..

[13] Martínez-Lage I., Martínez-Abella F., Vázquez-Herrero C., Pérez-Ordóñez J.L. (2012). Properties of plain concrete made with mixed recycled coarse aggregate. Construct. Build. Mater..

[14] Sabapathy L., Mohammed B.S., Al-Fakih A., Wahab M.M.A., Liew M.S., Amran Y.H.M. (2020). Acid and sulphate attacks on a rubberized engineered cementitious composite containing graphene oxide. Materials.

[15] Al-Fakih A., Mohammed B.S., Wahab M.M.A., Liew M.S., Mugahed Amran Y.H. (2020). Flexural behavior of rubberized concrete interlocking masonry walls under out-of-Plane load. Construct. Build. Mater..

[16] Lotfy A., Al-Fayez M. (2015). Performance evaluation of structural concrete using controlled quality coarse and fine recycled concrete aggregate. Cem. Concr. Compos..

[17] Hong D.L.H., Mohammed B.S., Al-Fakih A., Wahab M.M.A., Liew M.S., Mugahed Amran Y.H. (2020). Deformation properties of rubberized ecc incorporating nano graphene using response surface methodology. Materials.

[18] Agrawal D., Waghe U., Ansari K., Dighade R., Amran M., Qader D.N., Fediuk R. (2023). Experimental effect of pre-treatment of rubber fibers on mechanical properties of rubberized concrete. J. Mater. Res. Technol..

[19] Waghe U., Agrawal D., Ansari K., Wagh M., Amran M. (2023). Enhancing eco-concrete performance through synergistic integration of sugarcane , metakaolin , and crumb rubber : experimental investigation and response surface optimization. J. Eng. Res..

[20] Bravo M., De Brito J., Pontes J., Evangelista L. (2015). Mechanical performance of concrete made with aggregates from construction and demolition waste recycling plants. J. Clean. Prod..

[21] Zhou C., Chen Z. (2017). Mechanical properties of recycled concrete made with different types of coarse aggregate. Construct. Build. Mater..

[22] Ghorbel E., Wardeh G. (2017). Influence of recycled coarse aggregates incorporation on the fracture properties of concrete. Construct. Build. Mater..

[23] Bisht K., Ramana P.V. (2017). Evaluation of mechanical and durability properties of crumb rubber concrete. Construct. Build. Mater..

[24] Ganjian E., Khorami M., Akbar A. (2009). Scrap-tyre-rubber replacement for aggregate and filler in concrete. Construct. Build. Mater..

[25] Li N., Long G., Ma C., Fu Q., Zeng X., Ma K., Xie Y., Luo B. (2019). Properties of self-compacting concrete (SCC) with recycled tire rubber aggregate: a comprehensive study. J. Clean. Prod..

[26] Dhiraj Agrawal U., Waghe P., S.P.R (2021).

[27] Gholampour A., Ozbakkaloglu T., Hassanli R. (2017). Behavior of rubberized concrete under active confinement. Construct. Build. Mater..

[28] Agrawal Dhiraj, Waghe U.P., Goel M.D., Raut S.P., R.P (2023).

[29] Habib A., Yildirm U., Eren O. (2020). Mechanical and dynamic properties of high strength concrete with well graded coarse and fine tire rubber. Construct. Build. Mater..

[30] Rajhans P., Panda S.K., Nayak S. (2018). Sustainability on durability of self compacting concrete from C&D waste by improving porosity and hydrated compounds: a microstructural investigation. Construct. Build. Mater..

[31] Avudaiappan S., Prakatanoju S., Amran M., Aepuru R., Saavedra Flores E.I., Das R., Gupta R., Fediuk R., Vatin N. (2021). Experimental investigation and image processing to predict the properties of concrete with the addition of nano silica and rice husk Ash. Crystals.

[32] Lesovik V., Volodchenko A., Fediuk R., Mugahed Amran Y.H. (2021). Improving the hardened properties of nonautoclaved silicate materials using nanodispersed mine waste. J. Mater. Civ. Eng..

[33] Jayanthi V., Avudaiappan S., Amran M., Prakash K., Qader D.N., Ch M., Flores E.I.S., Rashid R.S.M. (2022). Innovative use of micronized biomass silica-GGBS as agro-industrial by-products for the production of a sustainable high-strength geopolymer concrete. Case Stud. Constr. Mater..

[34] Muthalvan R.S., Ravikumar S., Avudaiappan S., Amran M., Aepuru R., Vatin N., Fediuk R. (2021). The effect of superabsorbent polymer and nano-silica on the properties of blended cement. Crystals.

[35] Kisku N., Rajhans P., Panda S.K., Pandey V., Nayak S. (2020). Microstructural investigation of recycled aggregate concrete produced by adopting equal mortar volume method along with two stage mixing approach. Structures.

[36] Miller N.M., Tehrani F.M. (2017). Mechanical properties of rubberized lightweight aggregate concrete. Construct. Build. Mater..

[37] Su H., Yang J., Ling T., Ghataora G.S., Dirar S. (2015). Properties of concrete prepared with waste tyre rubber particles of uniform and varying sizes. J. Clean. Prod..

[38] Dong Q., Huang B., Shu X. (2013). Rubber modified concrete improved by chemically active coating and silane coupling agent. Construct. Build. Mater..

[39] Kardos A.J., Durham S.A. (2015). Strength, durability, and environmental properties of concrete utilizing recycled tire particles for pavement applications. Construct. Build. Mater..

[40] (2000). IS 456 plain and reinforced concrete - code of practice. IS 456-2000.

[41] (2019). IS 10262 concrete mix proportioning guidelines. IS 10262-2019.

[42] ASTM (2013). C138/C138M-13 standard test method for density (unit weight), yield, and air content (gravimetric). ASTM Int.

[43] (1959). IS 1199 methods of sampling and analysis of concrete. IS 1199.

[44] (2021). IS 516 hardened concrete - methods of test. IS 516-2021.

[45] (2011). BS 1881-122 BSI standards publication testing concrete Part 122 : method for determination of water absorption. BSI Stand.

[46] (2020). ASTM C1898 20 standard test methods for determining the chemical resistance of concrete products to acid attack. ASTM Int.

[47] Sharaky I., Issa U., Alwetaishi M., Abdelhafiz A., Shamseldin A., Al-Surf M., Al-Harthi M., Balabel A. (2021). Strength and water absorption of sustainable concrete produced with recycled basaltic concrete aggregates and powder. Sustain. Times.

[48] Warade H., Ansari K., Bhaskar K., Naaz Z., Khan M.A., Khan N.A., Zahmatkesh S., Hajiaghaei-Keshteli M. (2023). Optimizing the grass bio methanation in lab scale reactor utilizing response surface methodology. Biofuels.

[49] Ansari K., Shrikhande A., Malik M.A., Alahmadi A.A., Alwetaishi M., Alzaed A.N., Elbeltagi A. (2022). Optimization and operational analysis of domestic greywater treatment by electrocoagulation filtration using response surface methodology. Sustain. Times.

